# Distinguishing features of current COVID-19 vaccines: knowns and unknowns of antigen presentation and modes of action

**DOI:** 10.1038/s41541-021-00369-6

**Published:** 2021-08-16

**Authors:** Franz X. Heinz, Karin Stiasny

**Affiliations:** Center for Virology, Medical University of Vienna, Vienna, Austria

**Keywords:** RNA vaccines, Immunology

## Abstract

COVID-19 vaccines were developed with an unprecedented pace since the beginning of the pandemic. Several of them have reached market authorization and mass production, leading to their global application on a large scale. This enormous progress was achieved with fundamentally different vaccine technologies used in parallel. mRNA, adenoviral vector as well as inactivated whole-virus vaccines are now in widespread use, and a subunit vaccine is in a final stage of authorization. They all rely on the native viral spike protein (S) of SARS-CoV-2 for inducing potently neutralizing antibodies, but the presentation of this key antigen to the immune system differs substantially between the different categories of vaccines. In this article, we review the relevance of structural modifications of S in different vaccines and the different modes of antigen expression after vaccination with genetic adenovirus-vector and mRNA vaccines. Distinguishing characteristics and unknown features are highlighted in the context of protective antibody responses and reactogenicity of vaccines.

## Introduction

The development of COVID-19 vaccines was extremely fast and successful, with several manufacturers having obtained market authorization for their products within the first year from the identification of the virus (SARS-CoV-2). These vaccines are now used worldwide for mass immunization programs, and data on vaccine efficacies justify the hope that vaccination can indeed be the main instrument for preventing serious disease and death, and more generally for combating the pandemic^1–4^. Despite incompletely resolved questions (e.g. duration of immunity, prevention of transmission, and protection against emerging virus variants) the availability of effective COVID-19 vaccines is an enormous relief and certainly a great success story already now.

All current vaccines that are authorized for general use and for which clinical efficacy data have been published rely on the viral spike protein (S) as an immunogen, either alone or—in the case of inactivated virus vaccines—together with other viral proteins present in the viral particle (see sections below). Because of its essential functions during viral entry (receptor binding and membrane fusion), the S protein is the major target of antibodies that can potently neutralize the virus. Increasing evidence indicates that neutralizing antibodies are indeed a reliable correlate of protection^5–9^. The potency of these antibodies depends on high-affinity interactions with specific parts of the complex three-dimensional structure of the spike in a native conformation^10,11^. Efficient formation of such antibodies by B cells requires helper functions of CD4 T cells that are specifically stimulated by peptides derived from the same antigen in complex with MHCII molecules. Other components of cellular immunity, such as CD8 T cells, also contribute to immune responses after SARS-CoV-2 infection or vaccination, although their role in COVID-19 infections and protection from disease is still incompletely resolved^12,13^.

Current COVID-19 vaccines present the spike protein in very different ways to the immune system, and two main categories have to be discerned. The first category consists of mRNA and adenoviral vector vaccines (herein referred to as genetic vaccines, sections: “Genetic vaccines—general, “mRNA vaccines”, “Adenovirus-vector vaccines”), both of which do not contain the spike protein but provide genetic information for its biosynthesis in body cells of the vaccinee. With this kind of vaccines, the specific design of genetic sequences for the correct formation and presentation of properly folded spike proteins to B cells are in the foreground of interest. The second category encompasses protein-based approaches, i.e. classical inactivated whole-virus and innovative subunit vaccines, which contain S in different forms and combinations with adjuvants (Sections: “Protein-based vaccines—general”, “Inactivated vaccines”, “Subunit vaccines”). Irrespective of these categories, all vaccines have to cope with the intrinsic problem of conformational instability of the spike protein, whether it is synthesized in the vaccinee after genetic vaccination or in cell culture systems for production of conventional vaccines.

In this review, we discuss the biosynthesis and relevant structural features of the viral spike as a basis for understanding differences of its presentation in current COVID-19 vaccines. Our major focus is on variations of the constructs for S biosynthesis in genetic vaccines and on possible conformational differences of S in conventional vaccines. We also address the ‘grey matter’ of additional variables, such as ill-defined downstream production processes and purity of vaccines as well as differences in triggering sensors of innate immunity. All of these distinguishing features might provide clues to yet unresolved vaccine-specific determinants of immune responses, efficacy, and potentially adverse reactions. Our review is limited to those vaccines in current use for which phase 3 clinical efficacy data have been reported, and for which published information on the nature and manufacturing process exists. However, we would like to emphasize that there is an enormous pipeline of further developments (https://www.who.int/publications/m/item/draft-landscape-of-covid-19-candidate-vaccines), including subunit vaccines that contain only parts of the S protein, in some instances combined with components of other viral proteins. Therefore, the landscape of vaccines becoming available for general use may expand in the near future. Key features of the vaccines discussed in this review are summarized in Table 1.

**Table 1:** Vaccines discussed in this review.

Type	Manufacturer	Name	Stabilizing mutations	Virus strain	Eukaryotic production cell line	Dosage	References
mRNA	BioNTech-Pfizer (Germany, USA)	BNT162b2, Comirnaty	Yes (prolines)	Wuhan-Hu-1	not applicable	30 µg RNA (2x)	^44,46^
mRNA	Moderna-NIAID (USA)	mRNA-1273, COVID-19 Vaccine Moderna	yes (prolines)	Wuhan-Hu-1	not applicable	100 µg RNA (2x)	^45,47^
mRNA	CureVac (Germany)	CVnCoV	yes (prolines)	Wuhan-Hu-1	not applicable	12 µg RNA (2x)	^56^
Adenovector	University of Oxford-AstraZeneca (UK, Sweden)	COVID-19 vaccine AstraZeneca, AZD1222, ChAdOx1-S, Vaxzeria; Covishield	no	Wuhan-Hu-1	HEK293	5×10^10^ adenovirus vector particles (2x)	^65,66^
Adenovector	CanSino Biological Inc., Beijing Institute of Biotechnology (China)	Ad5 nCoV, Convidecia	no	Wuhan-Hu-1	HEK293	5×10^10^ adenovirus vector particles (2x)	^61^
Adenovector	Gamaleya Research Institute (Russia)	rAd26-S + rAd5-S, Gam-COVID-Vac, Sputnik V	no	Wuhan-Hu-1 (probably)	HEK293	10×10^10^ adenovirus vector particles (2x)	^67,68^
Adenovector	Janssen-Johnson & Johnson (NL/USA)	Ad26.COV2.S, COVID-19 Vaccine Janssen	yes (prolines, furin cleavage site)	Wuhan-Hu-1	PER.C6	5×10^10^ adenovirus vector particles (1x)	^19,62,63^
Inactivated whole virus	Sinopharm,Beijing Institute of Biological Products Co (China)	BBIBP-CorV, Sinopharm COVID-19 vaccine	not applicable	Wuhan-Hu-1-like HB02 strain	Vero	4 µg proposed (2x)	^89,90^
Inactivated whole virus	Sinovac (China)	CoronaVac	not applicable	Wuhan-Hu-1-like CN2 strain	Vero	3 µg proposed (2x)	^86,87^
Inactivated whole virus	Bharat Biotech (India)	Covaxin, BBV152	not applicable	NIV2020-770 (D614G)	Vero	6 µg proposed (2x)	^97,141,142^
Subunit	Novavax (USA)	NVX-CoV2373	yes (prolines, furin cleavage site)	Wuhan-Hu-1	Sf9	5 µg S ( +50 µg adjuvant) (2x)	^98,99^

## Biosynthesis and key properties of the spike protein

### Biosynthesis of S

**Fig. 1:**
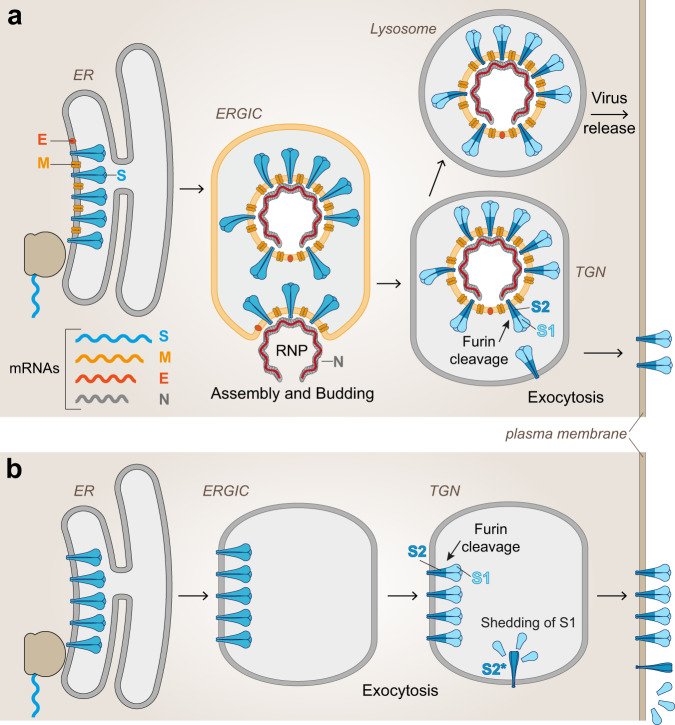
Biosynthesis and intracellular transport of S. **a** Infected cells: Subgenomic mRNAs for viral structural proteins are translated in association with the ER (S, M, and E) or in the cytoplasm (N), and virus assembly takes place in the ERGIC. Virus particles are transported through the TGN and released from the cells probably via lysosomes. During transport, S is cleaved into S1 and S2 by the cellular protease furin in the TGN. Some spike molecules, not assembled into virions, are also transported to the plasma membrane despite the presence of an ER retention signal^15^. **b** Transfected cells: Biosynthesis of S occurs in the absence of interactions with other viral proteins. Proteolytic cleavage into S1 and S2 occurs in the TGN similar to that in infected cells, but some shedding of cleaved S1 and conversion of S2 into its post-fusion structure (S2*) may occur in the absence of stabilizing mutations. ER—endoplasmic reticulum; ERGIC—endoplasmic reticulum Golgi intermediate compartment; TGN—Trans Golgi Network; RNP—Ribonucleoprotein; Viral proteins: S—spike, M—membrane; E—envelope; N—nucleoprotein.

In the course of cellular SARS-CoV-2 infection (Fig. 1a), the S protein is synthesized from one of the viral subgenomic mRNAs and co-translationally transported into the lumen of the endoplasmic reticulum (ER) by the use of a signal sequence at its N-terminus, comprising residues 1 to 13 of its total 1273 amino acids^14^. The signal sequence is cleaved off by signal peptidase attached to the inner ER membrane, generating the final N-terminus of the viral spike protein (14-QCVNL…). After completion of translation, the protein remains attached to the ER membrane through a C-terminal membrane anchor, trimerizes and moves to the ER-Golgi intermediate compartment (ERGIC) where virus assembly occurs by budding into the ERGIC lumen (Fig. 1a)^15^. During exocytosis, virus particles encounter the protease furin in the trans-Golgi network (TGN), which cleaves the S protein into its membrane-associated S2 subunit and the distal S1 subunit at a characteristic polybasic cleavage site^16^. These subunits remain associated in the trimer through noncovalent interactions, and the virus is probably secreted via exocytic lysosomes with disrupted lysosomal functions^17^. Extensive modifications by N- and O-glycosylation occur in the compartments encountered by S during its intracellular transport^18^.

When S is synthesized as an isolated protein (Fig. 1b) (as in mRNA and adenovirus vector vaccines as well as for production of recombinant subunit vaccines), the pathway of biosynthesis is very similar. However, the absence of interactions with other viral components for particle assembly may modulate glycosylation patterns and stability of the S trimers. Furthermore, S1 may dissociate from recombinantly produced spikes after furin cleavage in the TGN (shedding) and allow S2 to convert into its post-fusion conformation in the absence of mutations that remove the cleavage site^18^. Manipulations of the authentic viral signal sequence may cause inhomogeneities of the N-terminus and impair native folding of S^19^ (see also section “Adenovirus-vector vaccines” and Fig. 5).

### Structural properties of S

**Fig. 2:**
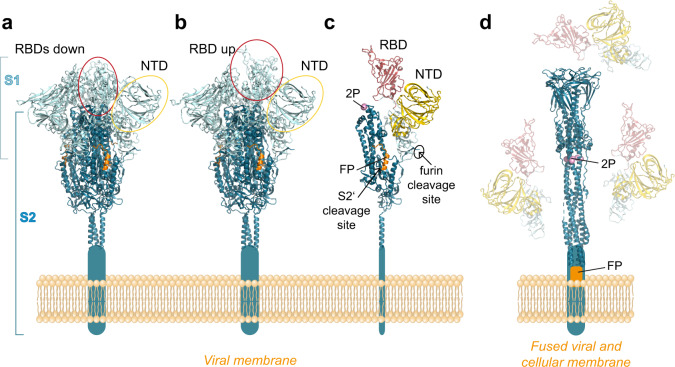
Structures of the spike protein in pre- and post-fusion conformations. **a** Trimeric pre-fusion spike with all RBDs in ‘down’ position. **b** Trimeric pre-fusion spike with one RBD in ‘up’ position. **c** Monomeric S protein of the pre-fusion spike with the RBD in red and NTD in gold, as well as the following structural details: The two stabilizing prolines (2 P) are shown in pink, the FP in orange. The two protease cleavage sites are indicated by arrows. **d** Trimeric post-fusion structure of S2, with the three dissociated S1 subunits, shaded in light colors. RBD—receptor binding domain; NTD—N-terminal domain; FP—fusion peptide. The structures were generated with PyMol, using protein data bank (PDB) files 7KRR and 7KRS^96^ for the pre-fusion forms, 6XRA for the post-fusion form^25^. The domains were colored according to reference.^20^.

Each monomer of S is composed of several structural elements, including the N-terminal domain (NTD) and receptor-binding domain (RBD) in S1, which occlude the S2 moiety in the native S trimer (Fig. 2a–c)^20,21^. The RBD oscillates between an ‘up’ and ‘down’ position, and interaction with the cellular receptor (ACE2) is only possible with the transiently exposed RBD in the up position^20,21^. In its mature form, the S trimer is metastable and ready to undergo triggered conformational changes that allow S2 to drive fusion of the viral and cellular membranes upon virus entry^22^. The trigger comprises binding of RBD to ACE2 and a further proteolytic cleavage by cellular proteases (in addition to the furin cleavage between S1 and S2) at the so-called S2’ site, resulting in the removal of a small sequence element and the exposure of the fusion peptide at the N-terminus of S2 (Fig. 2c)^22–24^. As a consequence of these changes, the S1 subunits dissociate from the trimer, releasing S2 from its constraints in the pre-fusion conformation to allow an irreversible conversion into a characteristic elongated post-fusion structure (Fig. 2d)^24–26^. The energy gained by the formation of this hairpin-like structure, in which the fusion peptide is juxtaposed to the C-terminal membrane anchor, is the driving force for viral membrane fusion during entry^22^.

The potential of the S trimer to adopt different conformations may pose a problem for its use in vaccines, because the native structure—required to induce potently neutralizing antibodies—may be disrupted during manufacturing of conventional vaccines or when the protein is expressed in cells of the vaccinee after genetic vaccination. Some manufacturers have therefore introduced stabilizing mutations that are intended to prevent inadvertent structural conversion of the labile S protein. These modifications (indicated in Table 1 and in the discussion of individual vaccines below) include two proline mutations in S2 (K986P and V987P) at the junction between two alpha helices in the pre-fusion form to avert their fusogenic conformational switch into a long alpha helix in the post-fusion form, and mutations that abolish furin cleavage between S1 and S2 to maintain the pre-fusion trimer and to prevent shedding of S1^18^ (Fig. 2c, d).

### Antigenic structure of S

A number of monoclonal antibodies were isolated from COVID-19 patients and used for antigenic characterization of the S trimer, including 3D structure determinations of complexes between S (or parts thereof) and antibody Fab fragments. Collectively, these data showed that the most potently neutralizing antibodies were specific for the RBD^27–34^, but several strongly neutralizing antibodies also recognized the NTD^27,34–37^, and some were dependent on the quaternary assembly of the trimer^27,38^. Neutralizing activity was also observed for antibodies against S2, but the potency was lower than of those against S1^27^. Importantly, the human neutralizing antibody response in SARS-CoV-2 infection appears to be dominated by RBD-specific antibodies, which—on average—were shown to contribute 90% of the total neutralizing activity of human post-infection sera^39^. It is therefore a major goal of all COVID-19 vaccines to present the spike and its RBD in a most native conformation for inducing a high proportion of potently neutralizing antibodies after vaccination.

## Vaccine-specific differences of S-antigen structure and presentation

The different classes of currently available COVID-19 vaccines exhibit fundamental differences with respect to their modes of action and the ways by which the spike antigen is presented to the immune system. In the following sections, we will discuss these basic differences, and provide information on variations and modifications that can affect the structural integrity of the spike in genetic and conventional vaccines.

### Genetic vaccines—general

The uniting feature of current genetic COVID-19 vaccines is the provision of mRNAs for the whole, membrane-anchored spike protein (Figs. 1, 2) in tissues after intramuscular application. RNA vaccines contain fully functional mRNAs that can be translated directly into the S protein, whereas additional biosynthetic steps are required with adenovirus vector vaccines, including intranuclear transcription of the vector DNA into RNA and processing to generate functional mRNAs. It is believed (but not systematically studied and formally shown) that muscle cells, fibroblasts, endothelial cells, and/or immune cells such as dendritic cells contribute to the expression of S after intramuscular vaccination^40–42^. Production of potently neutralizing antibodies requires the interaction of B cells with the native protein, most likely by recognition of the spike anchored in the plasma membrane of S-expressing cells (Fig.1b). In contrast, CD8 and CD4 T cells are stimulated by complexes of peptides (derived from intracellular S after its proteolytic processing) with MHCI and MHCII, respectively^43^.

### mRNA vaccines

The two mRNA vaccines in current widespread application (BioNTech-Pfizer and Moderna) (Table 1) are technologically very similar. They contain codon-optimized sequences for efficient expression of the full-length S protein and use the authentic signal sequence for its biosynthesis^44–47^ (Fig. 1b). Both constructs include the two stabilizing mutations in S2 (K986P and V987P) that were shown to prevent the conformational change of the pre-fusion into the post-fusion structure of S (section “Introduction” and Fig. 2c)^20,21^.

**Fig. 3:**
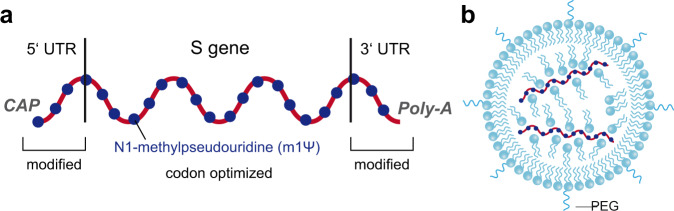
Configuration of mRNA vaccines. **a** Schematic of the vaccine mRNA in BionTech-Pfizer and Moderna vaccines. UTR—untranslated region. **b** Schematic of a lipidnanoparticle (LNP) used for delivery of mRNA vaccines. PEG—polyethyleneglycol.

The production process of vaccine mRNAs involves the cloning of the corresponding sequence into a plasmid DNA containing a DNA-dependent RNA-polymerase promoter. After amplification in bacterial cells, the plasmid DNA is linearized and impurities are removed before in vitro transcription into RNA. The addition of a 5′ cap structure is a critical part of this production step that has been improved by new technology suitable for large-scale production^48,49^. In vitro transcription is followed by several steps of mRNA purification, including the removal of dsRNA, which could lead to an excessive innate immune response and concomitant reactogenicity^48,50^. Both mRNA vaccines have modulated 5′ and 3′ untranslated sequences to optimize mRNA stability and translation efficiency^44,45^, and all uridines are replaced by N1-methylpseudouridine (m1Ψ) to further increase RNA stability and to reduce innate immune responses (Fig. 3a; see section “Vaccine-specific differences of innate responses”)^51,52^. Details of manufacturing processes may differ between the companies, and subtle product-specific variations of RNA sequences were recently confirmed by comparative analyses of RNA extracted from original vials of the two vaccines (https://github.com/NAalytics/Assemblies-of-putative-SARS-CoV2-spike-encoding-mRNA-sequences-for-vaccines-BNT-162b2-and-mRNA-1273/blob/main/Assemblies%20of%20putative%20SARS-CoV2-spike-encoding%20mRNA%20sequences%20for%20vaccines%20BNT-162b2%20and%20mRNA-1273.docx.pdf).

For delivery, the RNA vaccines are formulated as complexes with specific lipids in the form of lipid nanoparticles (LNP), which not only provide protection from RNA degradation in tissues but also facilitate cellular uptake and release into the cytoplasm for RNA translation (Fig. 3b)^53,54^. The components used for LNP formulation include phospholipids, cholesterol, special cationic (ionizable) lipids and polyethylene glycol (PEGylated) lipids that are mixed in a sophisticated and critical production step (parts of which are not documented in the published literature) to yield the final vaccine^53^. Specifically developed and improved ionizable lipids are used in the Moderna and Biontech-Pfizer vaccines (designated Lipid H, SM-102 and ALC-0315, respectively), which together with the molar ratios of the lipid components in LNPs play a critical role for RNA delivery^54^. The precise mechanisms of how the RNA is taken up by different cells after vaccination and escapes from LNPs and intracellular vesicles is incompletely resolved^53,55^. Collectively, there are subtle differences between the two vaccines, both with respect to the RNA and the LNP carriers, and a higher amount of RNA per dose is used in the Moderna vaccine (100 µg) than in the BioNTech-Pfizer vaccine (30 µg)^46,47^.

Another mRNA vaccine, manufactured by the company CureVac (current name CVnCoV; Table 1) is in an advanced stage of development^56^. The RNA in this vaccine is also codon-optimized and contains modifications to improve its performance, but—different from the BioNTech-Pfizer and Moderna mRNA vaccines described above—it does not contain the m1Ψ nucleoside modifications^57,58^. Recently, data from a phase 3 clinical trial became available, showing a relatively low efficacy of 47% at preventing disease (https://www.curevac.com/en/2021/06/16/curevac-provides-update-on-phase-2b-3-trial-of-first-generation-covid-19-vaccine-candidate-cvncov/), well below the efficacies reported for the BionTech-Pfizer and Moderna vaccines^46,47^ and below the requirement of at least 50% efficacy proposed by WHO (https://www.who.int/publications/m/item/considerations-for-the-assessment-of-covid-19-vaccines-for-listing-by-who). The low performance may be attributed in part to the high proportion of variants that have caused infections in the study population. The major problem, however, appears to reside in the relatively low dose of 12 µg RNA that had to be chosen to avoid intolerably strong side reactions in the absence of RNA modifications such as the m1Ψ nucleoside modifications used in the two authorized mRNA vaccines^59^. Results from a phase 1 clinical trial with the Curevac vaccine had indeed already shown relatively low titers of neutralizing antibodies induced by the dose used in the phase 3 clinical trial^56,59^.

Head-to-head comparisons of current mRNA vaccines with respect to possible differences in the efficiency of protein translation, stability or the stimulation of innate responses are not available in the literature. Persistence of RNA and its expression after different routes of application (including intramuscular) appears to be short (at least in mice), with a maximum of 10 days^60^.

### Adenovirus-vector vaccines

Compared to mRNA vaccines, adenovirus-vector vaccines comprise several additional layers of complexity (including production in mammalian cell cultures) that can lead to heterogeneities of immune reactions and adverse effects. Variations include (but are not limited to) the type of adenovirus used as a vector, genetic modifications of the vector, the cell lines used for vaccine production, procedures for purification, and the specific design of the gene for expressing S (Table 1).

Currently, four adenovirus-vector vaccines are in widespread use. These are the products (in alphabetical order) of CanSino Biological Inc./Beijing Institute of Biotechnology, Janssen-Johnson&Johnson, Oxford-AstraZeneca and The Gamaleya Institute Moscow (Table 1). They use derivatives of different adenoviruses as vectors for reasons more specifically discussed in section “Distinguishing features of vaccines independent of immunogen”, as follows: CanSino—human adenovirus 5^61^, Janssen-Johnson&Johnson—human adenovirus 26^19,62,63^, Oxford-AstraZeneca—chimpanzee adenovirus Y25^64–66^; Gamaleya Institute—human adenovirus 26 for the first vaccination and human adenovirus 5 for the second^67,68^.

**Fig. 4:**
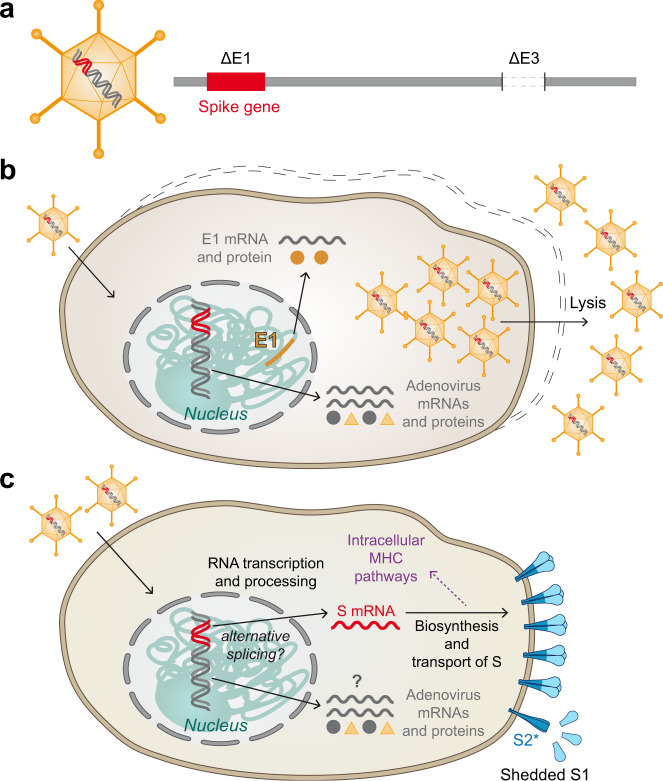
Principle of adenovirus vector vaccines. **a** Schematic of replication-incompetent adenoviral vector particle and its DNA. E1 and E3: Early adenovirus genes 1 and 3, respectively. **b** Formation of vaccine particles in production cell line complementing E1 from chromosomally integrated E1 gene. Release of newly produced vector particles through cell lysis. **c** Expression of spike in cells of vaccinated individuals. More or less shedding of S1 and conversion of S2 into its post-fusion structure (S2*) may occur in the absence of stabilizing mutations.

The unifying feature of all current adenovirus-vaccine vectors is the replacement of one of the early adenoviral genes (E1) for the full-length SARS-Cov-2 S gene in the adenoviral DNA (Fig. 4a) and the additional deletion of E3^19,61,62,64,65,69^. The loss of the E1 gene abolishes replication competence of the vector. Therefore, for production of the engineered particles as a vaccine, immortalized helper cell lines are used that contain the E1 gene in their chromosomal DNA and provide the missing function, allowing the biosynthesis of structural proteins, replication of modified genomic DNA, and finally assembly of replication-incompetent virus particles in the cells (Fig. 4b)^70^. Production cell lines for the Oxford-AstraZeneca, Gamaleya and CanSino vaccines are derived from primary human embryonic kidney cells (HEK293), and for the Janssen vaccine from human embryonic retinal cells (PER.C6) (Table 1). Quantitative recovery of adenoviral vector particles involves lysis of the cells by detergents (Fig. 4B, right) and further downstream processes for the removal of cellular components and free viral DNA^71^. Details of these processes, affecting the purity and quality of the final vaccines (containing at least 5 × 10^10^ particles per dose), are not accessible in the published literature (see section “Contaminations from cell substrates”).

Similar to mRNA vaccines, adenovirus vector vaccines are intended to result in the production of native S proteins from a specific mRNA in cells of the vaccinee (Figs. 1b, 4c). The pathway to this mRNA however is substantially more complex than with mRNA vaccines because it takes a detour of the adenoviral DNA through the nucleus (where it remains extrachromosomal) and requires a number of additional cellular processes, including RNA transcription and processing (Fig. 4c). Although in vitro model studies with one of the current adenovirus vector vaccines (ChAdOx1 nCoV-19; Table 1) have shown that S-coding transcripts dominate the transcription patterns, rare aberrant splicing or polyadenylation site usage were observed^72^. Recent work by Kowarz et al.^73^ provides further evidence for alternative splice events that might lead to the formation of C-terminally truncated and therefore soluble S protein. The authors speculate that such secreted forms may bind to ACE2-expressing endothelial cells and could contribute to thrombotic events via antibody-mediated mechanism as observed after vaccination with adenovirus vector COVID-19 vaccines^74,75^ (see also section “Reactions due to vaccine constituents other than the immunogen”).

In addition, background expression of remaining adenoviral genes has been demonstrated in this as well as in other studies with human adenovirus-based vectors^72,76^. It is part of the unknowns of current COVID-19 adenovirus vector vaccines, how the patterns of background-vector DNA and protein expression look like after vaccination and whether immune reactions to such proteins are induced.

Although the constructs for all four adenovirus-vector vaccines contain the full-length spike protein, there are some differences in construct design with respect to mutations for stabilizing S as well as to the signal peptide at the N-terminus of S that require attention. Only the Janssen vaccine contains S-stabilizing mutations (Table 1), comprising not only the two prolines in S2 but also the S1/S2 furin cleavage site, which is mutated from 682-RRAS-685 to SRAG^19,62^. Both modifications are intended to avoid conversion of S into the post-fusion structure (Fig. 2d)^19^.

**Fig. 5:**
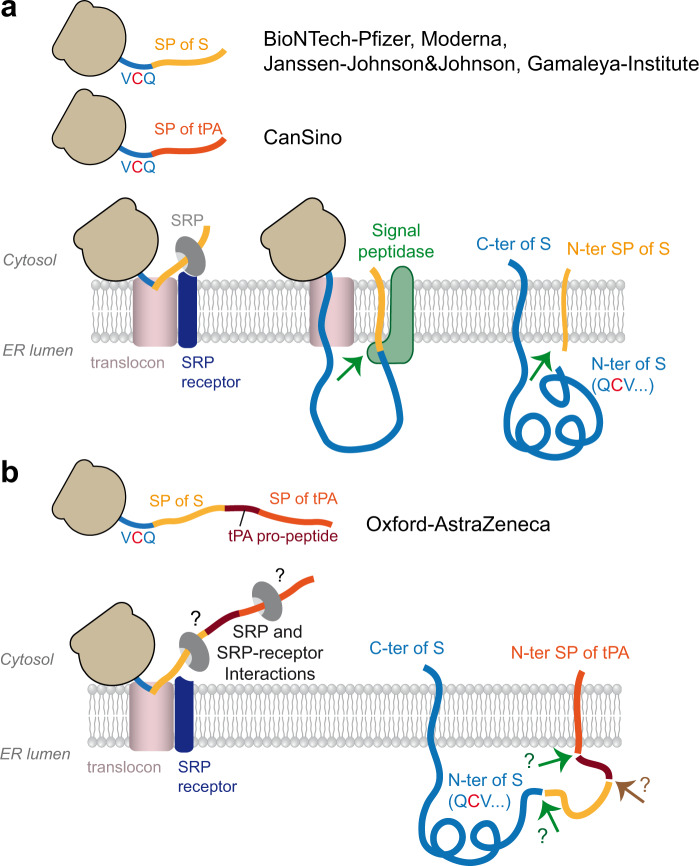
Signal sequence-mediated transport of S into the lumen of the ER. **a** Schematic of the process using the authentic viral signal peptide only (as in the vaccines of BionTech-Pfizer, Moderna, Janssen-Johnson&Johnson and Gamaleya Institute). In the CanSino vaccine, the signal peptide of S is replaced by that of human tPA (https://patents.google.com/patent/CN111218459B/en). **b** Schematic of the process using an additional N-terminal leader sequence (signal peptide and propeptide of tPA), as used in the vaccine of Oxford-Astra Zeneca, based on reference. ^80^). SP—signal peptide; SRP—signal recognition particle; tPA—tissue plasminogen activator; ER—endoplasmic reticulum; C-ter—C terminus; N-ter—N terminus.

Correct processing of the signal peptide by signal peptidase to generate the final N-terminus of S (Fig. 5a) may be especially critical for obtaining natively folded S, because there is a cysteine immediately downstream of the cleavage site (amino acid 2) that has to form an S-S bond with the cysteine at position 136. Janssen-Johnson&Johnson and Gamaleya-Institute use the authentic SARS-CoV-2 S protein signal sequence^19,67^, whereas CanSino replaced it with that of human tissue plasminogen activator (tPA) (Fig. 5a) (https://patents.google.com/patent/CN111218459B/en). In the Oxford-AstraZeneca vaccine, an extended form of the tPA signal sequence (containing the tPA propeptide) was engineered in front of the authentic S protein signal sequence^65,77,78^ (Fig. 5b), based on a previous study with Middle East respiratory syndrome coronavirus (MERS-CoV^79^ (Fig. 5b). Details of the engineered ‘leader sequence’ in the ChAdOX1-S vaccine are difficult to track, but likely comprise 32 to 34 amino acids of tPA (according to a document of the European Medicines Agency assessing thrombotic post-vaccination events, EMA/205598/2021) and may contain a P to A mutation at position 22 to improve processing by signal peptidase^80^. The extended N-terminal ‘leader sequence’ results in two consecutive signal peptides separated by an intervening stretch of tPA propeptide (Fig. 5b). This complex artificial sequence element may lead to some inhomogeneity in proteolytic processing and impairment of correct formation of the S N-terminus during biosynthesis, as recently shown in comparative model studies with similar constructs^19^.

Despite the absence of S2-stabilizing mutations, structural studies of the S protein expressed in HeLa cells from the Oxford-AstraZeneca ChAdOx1 nCoV-19 vaccine provided evidence for proper folding and presentation of the trimeric pre-fusion conformation at the cellular plasma membrane^78^. However, the authors discuss evidence of shedding of the cleaved S1 portion^78^, which has also been observed in model studies with unmodified S proteins compared to mutationally stabilized proteins^18^ (Fig. 2c). The effect of dissociation of soluble S1 from the trimer complex on the quality of immune responses is incompletely understood, but some data suggest it may contribute to a higher proportion of non-neutralizing relative to neutralizing antibodies^19,81^.

Animal experiments have shown that adenovirus-vector DNA can remain detectable for months after inoculation in transcriptionally active form^82^ in contrast to rapidly degraded RNA^55,83^. Persistence of antigen expression may be a distinctive feature of adenovirus vector vaccines, and has been proposed to contribute to induction of sustained immune responses and long-lasting immunity (reviewed in^41^).

### Protein-based vaccines—general

In parallel to genetic vaccines, more conventional approaches of vaccine development were pursued with similar intensity and led to the authorization of inactivated whole-virus vaccines and a subunit vaccine that is in a stage of pre-authorization (Table 1). Both of these technologies have already been applied successfully to the production of vaccines against other viral diseases^84^. With these vaccines—and in contrast to genetic vaccines—a predefined amount of the S immunogen/antigen is applied to the vaccinee, but—as discussed in the following sections—its conformational integrity may vary depending on the conditions used for vaccine preparation.

### Inactivated vaccines

Published information about the production process is available for two inactivated whole-virus vaccines manufactured by the Chinese companies Sinopharm and Sinovac (Table 1). In both instances, the virus is grown in Vero cells and inactivated by beta-propiolactone (BPL), which is used as an inactivating agent for other viral vaccines, including rabies vaccines^85^. Literature data indicate that production of the Sinovac vaccine includes several steps of virus purification, leading to a product that contains primarily the viral proteins and consists of essentially pure viral particles^86,87^. The degree of purity of the Sinopharm vaccine (referred to as BBIBP-CorV; Table 1) is less clear. Two pertinent references do not indicate specific steps of purification after inactivation and removal of cell debris^88,89^, but one figure in a publication by Wang et al.^90^ suggests a step of chromatography, albeit without providing details of this process and the purity of the vaccine. Both vaccines use aluminum hydroxide as an adjuvant. Another inactivated whole-virus vaccine using similar technology is produced by the Indian company Bharat and has received emergency use authorization in India even before completion of phase III clinical trials^91^ (Table 1). Details of purification processes used for the manufacturing of this vaccine are not available in published literature.

There are open questions concerning the structure of S in the inactivated vaccines. Electron microscopical pictures of the viral spikes in the Sinovac vaccine have been interpreted differently, either as displaying the pre-fusion structure^86^ or the post-fusion structure^25,92^. Several lines of evidence suggest that BPL-inactivation in combination with purification processes can indeed lead to the formation of the post-fusion spike and the concomitant dissociation of S1^25,92^. Preservation of the native trimeric pre-fusion conformation, in contrast, was observed in structural studies with formalin-inactivated virus^93^, suggesting that inactivation and/or purification procedures can influence the ratios of pre- and post-fusion conformations of S and thus the qualities of killed whole-virus vaccines. Such factors may contribute to variations in the efficacies reported in clinical trials with current inactivated whole-virus vaccines^94^. There is indirect evidence that virus strains having emerged later in the pandemic (e.g. containing the mutation D614G in S) may be more stable^95,96^ and therefore could serve as an improved substrate for the production of inactivated vaccines. The vaccine produced by Bharat is indeed based on a seed virus containing this mutation^97^ (Table 1).

### Subunit vaccines

So far, results of phase III clinical efficacy trials were reported and published for a single subunit vaccine only^98^, which is manufactured by the company Novavax (NVX-CoV2373) (Table 1)^98,99^ and is still in a stage before regulatory approval^100^. It consists of the trimeric full-length spike that is produced as a recombinant protein in insect Sf9 (*Spodoptera frugiperda*) cells using a baculovirus expression system and contains mutations to stabilize S2 (K986P and V987P) as well as to delete the furin cleavage site (682-RRAS-685 changed to QQAQ)^99^ (Table 1 and Fig. 2). The protein has its authentic membrane anchor and remains associated with the membranes of the Sf9 production cells. Therefore, isolation of the final product includes detergent solubilization of the cells and several steps of purification^101^. The company formulates the S trimer as a nanoparticle in polysorbate 80 (PS80) detergent and uses a special proprietary saponin-based adjuvant (Matrix-M™) that comprises 40 nm particles composed of Quillaja saponins, cholesterol and phospholipids^102,103^. High-resolution structural analysis revealed that the purified protein is stably locked in the preferred pre-fusion conformation, in part as free trimers and in part as multitrimer complexes^103^. The vaccine thus presents the correctly folded immunogen in essentially pure form and in combination with a potent adjuvant.

### Effectiveness of vaccines against viral variants

A plethora of viral mutants arose and spread since the emergence of SARS-CoV-2^104,105^. Some of these mutants are considered ‘Variants of Concern’ (VOCs) because of their highly efficient transmission, the concomitant replacement of previously circulating strains, and the presence of mutations in the spike protein that can lead to immune escape (https://www.who.int/en/activities/tracking-SARS-CoV-2-variants/). In principle, all current vaccines are affected similarly by VOCs, because they are all based on original wild-type strains from the early phase of the pandemic (see Table 1) and therefore their S protein sequences differ from those of VOCs to the same degrees. Indeed, substantially reduced neutralization titers against some VOCs were observed with sera after immunization with mRNA and adenovirus vector vaccines^106–109^, pointing to the importance of the problem. On the positive side, results of efficacy as well as field effectiveness studies in various countries using different vaccines indicated a high degree of protection also against circulating VOCs, in particular against the Alpha variant^2,98,110,111^. Prevention of infection with the Beta, Gamma and Delta variants might be lower, although evidence indicates substantial protection from severe disease after two vaccinations^110,112,113^. So far, no data are available that would allow a direct comparison of the various vaccines and their effectiveness against the different VOCs. Given the same antigenic difference of all vaccines relative to VOCs, the most important parameter determining cross-protection may be the quantity of neutralizing antibodies and relevant cellular immune reactivity at the time of infection. This quantitative aspect is important for future analyses of the impact of waning immunity on protection and decisions about optimal timings of booster immunizations. Efforts are also underway to replace existing vaccine strains and corresponding sequences for those of the most relevant circulating strains^114^.

## Distinguishing features of vaccines independent of immunogen structure

As outlined in the preceding sections, substantial differences appear to exist among current vaccines that can affect the conformation of S and its presentation to the immune system. Independent of such antigenic effects, the fundamentally different mechanisms of action and ways of production are likely to introduce additional variation to the characteristics of immune responses and possible adverse reactions. Adenovirus-vector and mRNA vaccines promote substantially different innate responses that will certainly influence the nature of adaptive immune responses^43^. There is evidence that the Oxford-AstraZeneca vaccine might induce higher levels of specific T cells, whereas mRNA vaccines might induce higher antibody titers^115–117^. The relevance of these differences for protection are not yet clear. Similarly, immune responses to protein-based vaccines are shaped by the adjuvant used, for example by shifting CD4 T cells towards either Th1 or Th2^118,119^. For meaningful conclusions, studies on these topics will require head-to-head comparisons of vaccines, and corresponding publications are expected to expand rapidly in the near future. Here, we briefly discuss existing data and describe distinguishing features that can contribute to differences among vaccine responses independent of the structure and presentation of the S immunogen.

### Contaminations from cell substrates

Contaminating cellular proteins can be present in all vaccines involving production in cell culture. This pertains to many well-used licensed vaccines such as those against influenza, measles and rabies^120^. The amount of impurities depends on the purification steps applied in the manufacturing process. Except for mRNA vaccines, different eukaryotic cell cultures are used in the production of current COVID-19 vaccines (see sections “Biosynthesis and key properties of the spike protein” and “Vaccine-specific differences of S-antigen structure and presentation” and Table 1). Constituents in the Oxford-AstraZeneca vaccine were recently analyzed in the context of a search for potential causes of *venous sinus thrombosis* as a rare post-vaccinal complication^121^. The study revealed that the vaccine contains vast numbers and amounts of cellular proteins from the human HEK293 production cell line, in addition to adenoviral proteins and the S protein, which is apparently also synthesized already during the manufacturing process. The total amount of protein per dose was found to be 35 to 40 µg, most of which can be assumed to be cellular protein, because the protein of 5 × 10^10^ adenovirus particles per dose would account for only about 8 µg (for calculation see^122^). Although corresponding data do not yet exist in the public domain for the other adenoviral vector vaccines, the problem of cellular impurities may be similar, because they all depend on the lysis of production cells for releasing the engineered vector particles (section “Adenovirus-vector vaccines”). Details of purification procedures during manufacturing of the current adenovirus vector vaccines may differ but are not published.

Cell cultures are also used for production of the inactivated whole-virus vaccines (Vero cells) of Sinopharm^88^, Sinovac^86^ and Bharat^97^ as well as for the Novavax subunit vaccine (insect Sf9 cells)^99,101^ (sections “Inactivated vaccines” and “Subunit vaccines” and Table 1). According to published literature, manufacturing of the Sinovac and Novavax vaccines involves extensive purification procedures^86,101^, suggesting that the antigenic contents of these products consists primarily of the proteins of the virus particle or the isolated spike trimer, respectively. Details of purification procedures of the Bharat vaccine and the degree of cellular contaminants in the Sinopharm vaccine (which is less purified than that of Sinovac according to ref. ^88^) could not be found in the literature.

### Reactions due to vaccine constituents other than the immunogen

Vaccination of millions or even billions of people within a short time window allows identification of rare adverse reactions that would otherwise be difficult to be linked causally to vaccination. Currently, a slightly but significantly increased risk of thrombotic events (including cerebral venous sinus thrombosis) was reported after vaccination with Oxford-AstraZeneca and Janssen adenovirus vector vaccines and has raised considerable concern^74,75^. Collectively, this kind of adverse event is designated ‘vaccine-induced immune thrombotic thrombocytopenia’ (VITT). Sophisticated analyses of the ChAdOx1 nCoV-19 vaccine to elucidate underlying pathogenic mechanisms suggest that constituents such as viral DNA and/or cellular proteins can favor the formation of antibodies against platelet factor 4 (PF4), thus promoting VITT^74,121^. Information on cellular impurities are so far restricted to ChAdOx1 and comparative analyses of all adenovector vaccines are not yet available.

After vaccination with mRNA vaccines, rare events of anaphylactic shock above the average incidence in the population have been reported, largely in individuals with a history of allergy^123,124^. Most of the allergens are proteins, which are not contained in these chemically defined vaccines (section “mRNA vaccines”). One of the constituents discussed as being causally linked to anaphylaxis is polyethylene glycol (PEG), which is used in the formulation of LNPs that protect the RNA and facilitate its transfer into cells (section “mRNA vaccines”). It has been speculated that pre-existing PEG antibodies might be involved in these allergic events^124^. Corresponding scientific investigations into the mechanisms of vaccine-induced anaphylactic reactions are ongoing^125^.

### Vector immunity

Effects of pre-existing and vaccination-induced immunity against the vector are a special feature of adenovirus vector vaccines. High rates of seropositivity against adenovirus 5 (the pioneer of adenovirus vector development) have been reported in the population^126,127^, and a number of studies have shown that pre-existing vector immunity can impair the response to the vaccine antigen^128–130^. Adenovirus 5 is used in the CanSino vaccine and the second dose of Gamaleya vaccines (section “Adenovirus-vector vaccines” and Table 1). For reducing potential negative effects of pre-existing immunity, alternative adenoviruses were developed as vectors, one of them adenovirus 26, which has lower rates of seropositivity in the population^127^ and is now used in the Janssen-Johnson&Johnson vaccine^19,62^ as well as in the first shot of the Gamaleya-Institute vaccine^67,68^. These considerations of vector immunity also prompted the development of non-human adenovirus vectors such as ChAdOx1 derived from chimpanzee adenovirus Y25^64^, now used in the Oxford-AstraZeneca vaccine^77^. In this case, seropositivity is negligible in Europe (zero in the UK,^64^) and low in Africa (9% in Gambian adults,^64,131^).

Irrespective of pre-existing immunity, all adenovirus vector vaccines are prone to induce immune responses against the vector particles^129^. Each dose contains 5 × 10^10^ or 10 × 10^10^ adenoviral particles (Table 1), which corresponds to 8 or 16 µg of adenoviral protein (for calculation see ref. ^122^). It is unclear, at present, which influences anti-vector responses will have on necessary COVID-19 booster vaccinations in the future. Possible remedies are prime-boost regimens as already used for vaccination with the Gamaleya-Institute vaccine (Ad26 followed by Ad5) or combinations with other classes of vaccines such as mRNA vaccines. Corresponding studies are in progress (Com-Cov study: Oxdorf-AstraZeneca and BionTech-Pfizer, launched in February^132^).

### Vaccine-specific differences of innate responses

Specific features of adaptive immune responses are strongly influenced and shaped by innate responses that are triggered by pathogen-associated molecular patterns (PAMPs) and their sensing by pattern recognition receptors (PRRs) (reviewed in ref. ^133^). Current COVID-19 vaccines are very different with respect to their compositions and modes of action, and therefore vaccine-induced innate responses will vary considerably. Adenoviral vectors contain PAMPs that can be sensed by TLRs at the plasma membrane (TLR2 and TLR4) and the endosomally located TLR9 (reviewed in ref. ^41^). In addition, the viral DNA itself can be sensed after endosomal rupture by cytosolic DNA sensors such as cGAS and the inflammasome, resulting in downstream signaling cascades for producing antiviral factors such as type I interferons^41^.

Innate responses to RNA that enters cells from the outside (such as in RNA virus infections or mRNA vaccination) differ from those stimulated by adenoviruses, because RNA is sensed by other PRRs, including TLR3, TLR7 and TLR8, all located in endosomes^134,135^. Sensors in the cytoplasm, such as retinoic-inducible gene I (RIG-I) and melanoma differentiation-associated antigen 5 (MDA-5) recognize preferentially dsRNA, also leading to stimulation of type I IFN secretion^134,135^. Excessive innate responses can not only result in strong reactogenicity of vaccination but also restrict antigen translation from the vaccine RNA, thus impairing adaptive immune responses. In the BionTech-Pfizer and Moderna vaccines this problem was taken into account by modifications of the RNA sequence and the inclusion of m1Ψ (section “mRNA vaccines”), which is not contained in CureVac’s mRNA vaccine^56^. In addition to direct triggers of innate immunity by RNA, other constituents of LNPs can contribute to vaccine-induced inflammatory reactions and provide adjuvant activity for adaptive immune responses. Such effects have been specifically shown for the ionizable lipid component in LNPs^124^. Head-to-head comparisons of mRNA vaccines will be informative to identify and evaluate differences of innate and adaptive responses as well as reactogenicity between representatives of this class of COVID-19 vaccines.

Due to their capacity to stimulate innate responses, the genetic vaccines are referred to as being ‘self-adjuvanted’^55,136^. Protein-based vaccines such as inactivated whole-virus vaccines or subunit vaccines are usually not sufficiently immunogenic on their own and require the addition of adjuvants. Alum is the most frequently used adjuvant in human vaccines and is used in the Sinopharm and Sinovac vaccines^137,138^. This adjuvant results in polarization towards a Th2 response, which has been regarded as unfavorable in the case of coronavirus and other viral infections and vaccinations^118,139,140^. Therefore, other adjuvants or combinations thereof with Alum have been developed for use in COVID-19 vaccines^138^. The inactivated whole virus vaccine produced by Bharat (Covaxin, Table 1) is adjuvanted with an imidazoquinoline class molecule (IMDG, a TLR 7/8 agonist) adsorbed on aluminum hydroxide gel (Algel-IMDG) that shifts the response towards Th1^97,141,142^. Another BPL-inactivated whole-virus vaccine in development (by the European company Valneva) makes use of Alum in combination with CpG to induce preferentially a desired Th1 response^138^, and a similar effect has been attributed to the Matrix-MTM adjuvant used in the Novavax subunit vaccine^99,101,102^.

## Conclusions

The severe consequences of the COVID-19 pandemic have created a pressing need for vaccines that not only prevent serious disease but preferentially also transmission. Several of the 291 candidates listed in the COVID-19 vaccine pipeline by WHO (184 pre-clinical and 107 in clinical development) (https://www.who.int/publications/m/item/draft-landscape-of-covid-19-candidate-vaccines, accessed on July 9, 2021), have already reached the market and are used for mass immunization. They all proved to exceed initial hopes and maximal expectations of 50 % protection^143,144^, displaying efficacies in preventing clinical disease of more than 90% in certain instances. Although all current vaccines for which phase 3 efficacy data are available rely on the whole viral spike protein as an antigen, its presentation to the immune system is strikingly different not only between genetic vaccines and protein-based vaccines, but also between vaccines within these categories. In addition, approaches to cope with the problem of the lability of the viral S protein cause variation across all current vaccines. These also differ with respect to their degree of purity (presence of extraneous proteins from the production process) and other vaccine constituents that can affect immune responses and cause adverse events. We have reviewed the most apparent and significant differences among the vaccines as far as they can be recognized from published literature, which unfortunately is still incomplete. Hopefully, more details will become available in the near future. Comparative analyses of antibody and T cell responses and their fine specificities will allow indirect but important conclusions to be drawn. Studies are emerging that address antibody formation to the different domains of S and analyze the ratio of neutralizing and non-neutralizing antibodies as an important parameter of vaccine performance^145,146^. These data can serve as an indirect measure for the structural integrity of S in the vaccines and the quality of B cell immune responses. Head-to-head comparisons of vaccinated cohorts will be especially insightful, considering the profound differences of antigen presentation and principles of action of current COVID-19 vaccines.
